# Epicardial hematoma due to heart positioner device in minimally invasive coronary artery bypass

**DOI:** 10.1093/jscr/rjae417

**Published:** 2024-06-19

**Authors:** Nobuhiro Mochizuki, Ryohei Ushioda, Dit Yoongtong, Boonsap Sakboon, Jaroen Cheewinmethasiri, Hiroyuki Kamiya, Nuttapon Arayawudhikul

**Affiliations:** Cardiovascular and Thoracic Surgery Unit, Department of Surgery, Lampang Hospital, Lampang, Thailand; Department of Cardiac Surgery, Asahikawa Medical University, Asahikawa, Midorigaoka 1-1-1, Japan; Cardiovascular and Thoracic Surgery Unit, Department of Surgery, Lampang Hospital, Lampang, Thailand; Department of Cardiac Surgery, Asahikawa Medical University, Asahikawa, Midorigaoka 1-1-1, Japan; Cardiovascular and Thoracic Surgery Unit, Department of Surgery, Lampang Hospital, Lampang, Thailand; Cardiovascular and Thoracic Surgery Unit, Department of Surgery, Lampang Hospital, Lampang, Thailand; Cardiovascular and Thoracic Surgery Unit, Department of Surgery, Lampang Hospital, Lampang, Thailand; Department of Cardiac Surgery, Asahikawa Medical University, Asahikawa, Midorigaoka 1-1-1, Japan; Department of Cardiac Surgery, Asahikawa Medical University, Asahikawa, Midorigaoka 1-1-1, Japan; Cardiovascular and Thoracic Surgery Unit, Department of Surgery, Lampang Hospital, Lampang, Thailand

**Keywords:** off-pump coronary artery bypass grafting, minimally invasive cardiac surgery, epicardial hematoma, heart positioner

## Abstract

Minimally invasive cardiac surgery off-pump coronary artery bypass (MICSOPCAB) has become increasingly prevalent, with devices like the heart positioner aiding in surgical precision. However, rare complications such as epicardial hematoma can occur. Here, we present a case of a 75-year-old man undergoing MICSOPCAB who developed an epicardial hematoma due to the heart positioner. The hematoma was successfully repaired intraoperatively with direct suturing and large felts. Postoperative recovery was uneventful, highlighting the importance of vigilant monitoring and prompt management of such complications. This case underscores the need for careful attention during the use of cardiac positioners to minimize adverse events and ensure favorable patient outcomes.

## Introduction

We have performed ~300 cases of minimally invasive cardiac surgery off-pump coronary artery bypass (MICSOPCAB) from August 2017 to April 2024. The left intercostal approach enables easier access for making left internal thoracic artery (LITA) - left anterior descending (LAD) artery. However, there are some tips for exposing the lateral and posterior wall. In our off-pump coronary artery bypass (OPCAB) cases, the heart positioner device is commonly utilized for anastomosis to the circumflex artery and the right coronary artery regions. Significant complications associated with the device include epicardial hematoma, although reports of such occurrences are extremely rare [1, 2]^.^ Here, we report a case of epicardial hematoma caused by the heart positioner in a patient undergoing MICSOPCAB, along with the repair method.

## Case presentation

**Figure 1:**
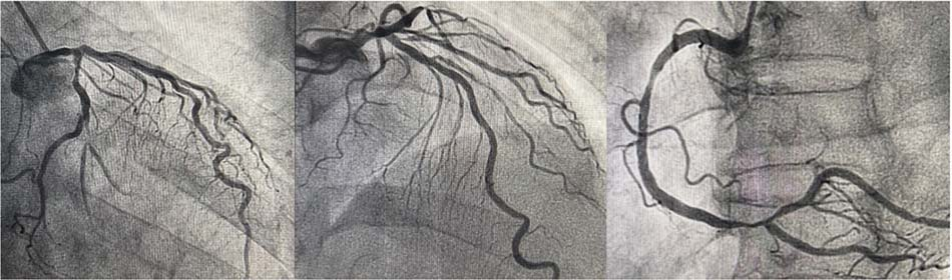
This coronary angiography indicates triple-vessel coronary artery disease. (LMT: 50% stenosis, LAD proximal: 90% stenosis, OM2: 80% stenosis, RCA distal: 90% stenosis)

The patient was a 75-year-old man with a non-ST elevation myocardial infarction. His underlying diseases were hypertension, dyslipidemia, and chronicle smoking. Coronary angiography revealed triple coronary artery disease with left main trunk (Fig. 1). His transthoracic echocardiography showed no valvular disease, normal left ventricular (LV) ejection fraction (EF) of 64% and left ventricular enlargement (LV end-diastolic diameter [LVDd]; 6.8 cm). In conclusion, He was referred for MICSOPCAB.

**Figure 2:**
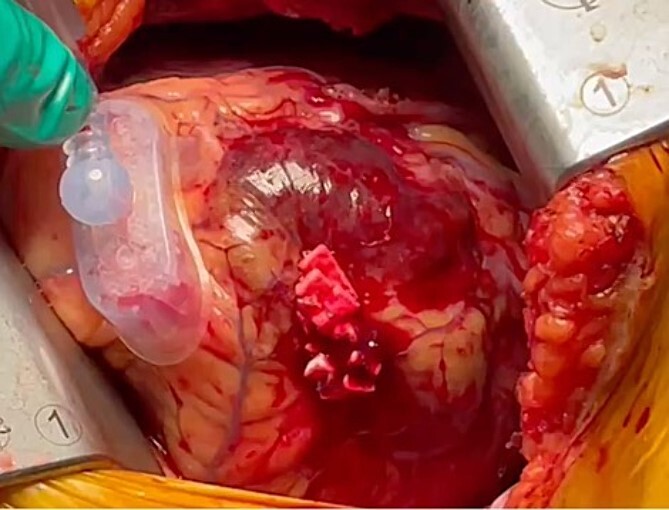
We observed an epicardial hematoma at apex of the left ventricular when we detached one of the Tentacles NEO suction cups.

**Figure 3:**
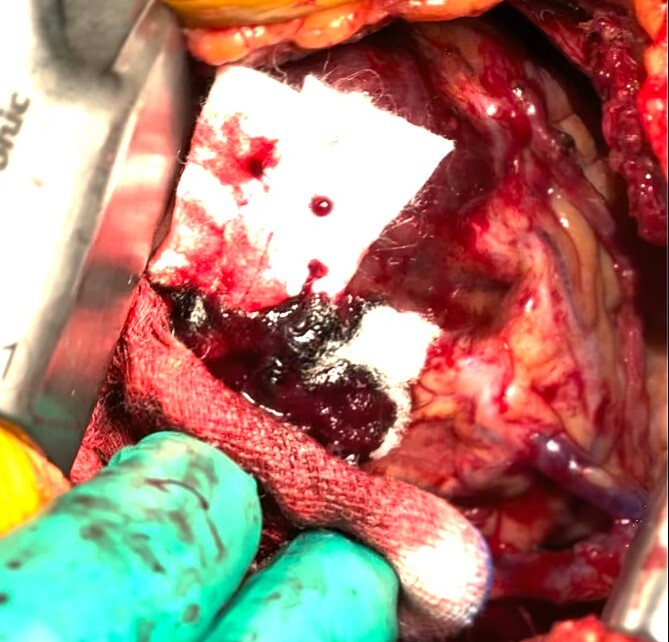
The bleeding spot was repaired using a continuous suture with 4–0 prolene and two large felts. Following the administration of protamine, effective hemostasis was achieved.

The patient was positioned by elevating in a 30° to 40° right lateral decubitus position to facilitate a widened intercostal space. The patient was intubated with a double-lumen endotracheal tube. A 10 cm left mini-thoracotomy was performed in the fourth intercostal space, with 1/3 of the incision medial to the mid-clavicular line. The ThoraTrak® MICS Retractor System (Medtronic Inc., MN, USA) was employed to achieve optimal access to the left chest and effectively visualize the internal mammary arteries. The LITA was harvested in a skeletonized fashion under direct vision, and the saphenous vein (SVG) was harvested from the right leg. To maintain the activated clotting time > 280 s, heparin (1 mg/kg) was administered after the LITA harvest. In this case, the Tentacles NEO (Sumitomo Bakeride, Akita, Japan) was used as a heart poisoner and cardiac stabilizer. We made four distal anastomoses (LITA-LAD, Aorta-SVG1-first diagonal, Y-composite SVG2-OM- posterolateral artery). When one of the suction cups attached to the apex of LV was detached, an epicardial hematoma was coming at the attachment site (Fig. 2). This bleeding point was repaired by continuous suture by 4–0 prolene with two big felt. After protamine was given, good hemostasis was achieved (Fig. 3).

After the operation, the patient was extubated within 24 hours. The coronary computed tomography angiography (CTA) was performed on the fourth day. All grafts were patent, and there was no left ventricular aneurysm at the bleeding point. The postoperative course was uneventful, and the patient was discharged on the fifth day after the operation.

## Discussion

To the best of our knowledge, this is the first report describing iatrogenic epicardial hematoma caused by the Tentacle heart positioner. Heart positioners facilitate optimal cardiac exposure during OPCAB, suctioning the surface of the heart like stabilizer devices. Especially, the Tentacles Neo heart positioner is highly useful in the MICSOPCAB procedure in limited working spaces because three small suction cups with flexible traction cords can be pulled in any direction, facilitating the appropriate positioning of the heart [3]. In our hospital, it has frequently been used during MICSOPCAB procedures. Epicardial injury is a rare complication associated with the use of cardiac positioners, with reported cases being limited [1, 2]. The Tentacle NEO’s suction pressure is comparable to that of the other suction devices, ranging from −200 to −400 mmHg [1, 2, 4]. Therefore, meticulous attention is required when manipulating the device to avoid excessive traction on the heart.

Epicardial bleeding or hematoma has frequently been reported as a complication of percutaneous coronary intervention (PCI) [5]. Vessel wall penetration with guide wires, balloon inflation in the subintima, or overexpansion of the coronary artery by an oversized balloon can induce coronary perforation and rupture, potentially leading to epicardial bleeding or hematoma. On the other hand, in our case, external pressure damage to the vessels in the suction cup area caused the epicardial hematoma. A hematoma compressing the surrounding coronary arteries or the heart itself can cause myocardial ischemia or cardiac tamponade [6–8]. Even with prompt repair, bad postoperation courses are often observed due to the effects of it [4–6]. In our case, repairing the injury during this operation was fortunate, likely averting potential complications from the hematoma.

Previous reports have documented surgical techniques for treating epicardial bleeding or hematoma, including direct suturing and epicardial patching with pericardium from human or bovine sources [1, 2, 4]. Mandke attempted to seal the large area of injured epicardium associated with the Octopus 3 stabilizer by using an in situ autologous pericardial patch held in position [1]. Ariyama et al. presented a case of epicardial bleeding caused by the Starfish’s suction cups (Medtronic, Inc, Minneapolis, MN, USA), which was successfully controlled with compression using Tachocomb (Nycomed, Zurich, Switzerland) due to venous bleeding [2]. Similarly, it was reported that complete hemostasis at the site of injury after PCI was achieved by attaching fibrin membranes [6]. In this case, we successfully achieved hemostasis by applying direct continuous sutures with two large felts. However, epicardial bleeding or hematoma may occur delayed, potentially manifesting after pericardial or chest closure. Moreover, there remains a possibility of recurrent bleeding even after repair [1, 2]. Therefore, diligent follow-up monitoring remains essential even after bleeding has been controlled in the operating room. For example, we should pay attention to postoperative cardiac tamponade signs such as central venous pressure elevation. Our hospital routinely performs postoperative CTA to check graft patency and pericardial effusion before discharge in MICSOPCAB cases.

## Conclusion

We successfully managed the repair of an epicardial hematoma due to the Tentacles Neo heart positioner. Given the potential for epicardial bleeding and hematoma leads to significant postoperative complications, cautious follow-up is essential.
